# Supplementary material for: Small molecule cognitive enhancer reverses age-related memory decline in mice
Source: eLife. 2020 Dec 1;9:e62048. doi: 10.7554/eLife.62048 (PMC7721440; doi:10.7554/eLife.62048)
Supplement: Figure 3—source data 1. — Column 1 – reagent name; Column 2 – Company of purchase; Column 3 – Catalog number. [file elife-62048-fig3-data1.docx]

**Figure 3 – Source Data 1. List of Electrophysiology Reagents**.

| **REAGENTS FOR PATCHING** | **Company** | **Product#** |
| --- | --- | --- |
| Sucrose | Sigma-Aldrich, St. Louis, MO | S5016 |
| NaH_2_PO_4_ | Sigma-Aldrich, St. Louis, MO | S9638 |
| NaHCO_3_ | Sigma-Aldrich, St. Louis, MO | S6014 |
| KCl | Sigma-Aldrich, St. Louis, MO | P9333 |
| NaCl | Sigma-Aldrich, St. Louis, MO | S9888 |
| CaCl_2_ | Sigma-Aldrich, St. Louis, MO | 223506 |
| MgCl_2_ | Sigma-Aldrich, St. Louis, MO | M9272 |
| Dextrose | Sigma-Aldrich, St. Louis, MO | G5767 |
| Ascorbic acid | Sigma-Aldrich, St. Louis, MO | A5960 |
| Sodium pyruvate | Sigma-Aldrich, St. Louis, MO | P5280 |
| Potassium gluconate | Sigma-Aldrich, St. Louis, MO | P1847 |
| HEPES | Sigma-Aldrich, St. Louis, MO | H3375 |
| MgATP | Sigma-Aldrich, St. Louis, MO | A9187 |
| Na_3_GTP | Sigma-Aldrich, St. Louis, MO | G8877 |
| 2K-phosphcreatine | Millipore, Burlington, MA | 237911 |
| Biocytin | Tocris, Bristol, UK | 3349 |
