# Supplementary material for: Small molecule cognitive enhancer reverses age-related memory decline in mice
Source: eLife. 2020 Dec 1;9:e62048. doi: 10.7554/eLife.62048 (PMC7721440; doi:10.7554/eLife.62048)
Supplement: Transparent reporting form [file elife-62048-transrepform.docx]

***eLife’s* transparent reporting form**

We encourage authors to provide detailed information *within their submission* to facilitate the interpretation and replication of experiments. Authors can upload supporting documentation to indicate the use of appropriate reporting guidelines for health-related research (see [EQUATOR Network](http://www.equator-network.org/%20)), life science research (see the [BioSharing Information Resource](https://biosharing.org/)), or the [ARRIVE guidelines](http://www.plosbiology.org/article/info:doi/10.1371/journal.pbio.1000412) for reporting work involving animal research. Where applicable, authors should refer to any relevant reporting standards documents in this form.

If you have any questions, please consult our Journal Policies and/or contact us: [editorial@elifesciences.org](mailto:editorial@elifesciences.org).

**Sample-size estimation**

- You should state whether an appropriate sample size was computed when the study was being designed
- You should state the statistical method of sample size computation and any required assumptions
- If no explicit power analysis was used, you should describe how you decided what sample (replicate) size (number) to use

Please outline where this information can be found within the submission (e.g., sections or figure legends), or explain why this information doesn’t apply to your submission:

The numbers of mice used was sufficient to result in statistically significant differences using standard power calculations with alpha = 0.05 and a power of 0.8. We used an online tool (<http://www.bu.edu/orccommittees/iacuc/policies-and-guidelines/sample-size-calculations/)> to calculate power and samples size based on experience with the respective tests, variability of the assays and inter-individual differences within groups. All experiments were randomized and blinded by an independent researcher prior to treatment.

This information is found in the METHODS section, Statistical Analysis subheading of the manuscript.

**Replicates**

- You should report how often each experiment was performed
- You should include a definition of biological versus technical replication
- The data obtained should be provided and sufficient information should be provided to indicate the number of independent biological and/or technical replicates
- If you encountered any outliers, you should describe how these were handled
- Criteria for exclusion/inclusion of data should be clearly stated
- High-throughput sequence data should be uploaded before submission, with a private link for reviewers provided (these are available from both GEO and ArrayExpress)

Please outline where this information can be found within the submission (e.g., sections or figure legends), or explain why this information doesn’t apply to your submission:

Methodology of how data is obtained can be found in the following sections: METHODS, FIGURE LEGENDS and RESULTS.

Data is presented as dot plots to allow for readers to visualize individual animals/tissues/cells. Distinctions are made in the FIGURE LEGENDS.

The following statement is found in the METHODS section, Statistical Analysis subheading of the manuscript.: ‘Biological replicates are measurements of biologically distinct animals/tissues/cells used to measure biological differences. Technical replicates are repeated measurements of the same animals/tissues/cells.’

The following statement is found in the METHODS section, Statistical Analysis subheading of the manuscript: ‘Group outliers were determined (GraphPad Software Outlier Test-Grubb’s test) and excluded from analysis. At most a single animal was excluded from each experimental cohort.’

**Statistical reporting**

- Statistical analysis methods should be described and justified
- Raw data should be presented in figures whenever informative to do so (typically when N per group is less than 10)
- For each experiment, you should identify the statistical tests used, exact values of N, definitions of center, methods of multiple test correction, and dispersion and precision measures (e.g., mean, median, SD, SEM, confidence intervals; and, for the major substantive results, a measure of effect size (e.g., Pearson's r, Cohen's d)
- Report exact p-values wherever possible alongside the summary statistics and 95% confidence intervals. These should be reported for all key questions and not only when the p-value is less than 0.05.

Please outline where this information can be found within the submission (e.g., sections or figure legends), or explain why this information doesn’t apply to your submission:

Statistical (test used, n, mean, SEM, summary statistics, significance levels) reporting is found in the FIGURE LEGENDS and METHODS section.

Figure data is presented as dot plots so raw values can be observed.

(For large datasets, or papers with a very large number of statistical tests, you may upload a single table file with tests, Ns, etc., with reference to sections in the manuscript.)

**Group allocation**

- Indicate how samples were allocated into experimental groups (in the case of clinical studies, please specify allocation to treatment method); if randomization was used, please also state if restricted randomization was applied
- Indicate if masking was used during group allocation, data collection and/or data analysis

Please outline where this information can be found within the submission (e.g., sections or figure legends), or explain why this information doesn’t apply to your submission:

The following statement is included in the METHODS section: ‘For all assays the experimenter(s) were blinded to therapeutic intervention. Prior to behavioral analysis animals were inspected for gross motor impairments. Animals were inspected for whisker loss, limb immobility (included grip strength) and eye occlusions. If animals displayed *any* of these impairments, they were excluded.’

**Additional data files (“source data”)**

- We encourage you to upload relevant additional data files, such as numerical data that are represented as a graph in a figure, or as a summary table
- Where provided, these should be in the most useful format, and they can be uploaded as “Source data” files linked to a main figure or table
- Include model definition files including the full list of parameters used
- Include code used for data analysis (e.g., R, MatLab)
- Avoid stating that data files are “available upon request”

Please indicate the figures or tables for which source data files have been provided:

No large datasets were used.
